# Indirect facilitation between prey promotes asymmetric apparent competition

**DOI:** 10.1111/1365-2656.13768

**Published:** 2022-07-10

**Authors:** Nicholas S. Lorusso, Cara A. Faillace

**Affiliations:** ^1^ Department of Ecology, Evolution, and Natural Resources Rutgers University New Brunswick New Jersey USA; ^2^ Department of Life Sciences University of North Texas at Dallas Dallas Texas USA; ^3^ University of Pittsburgh Pittsburgh Pennsylvania USA

**Keywords:** apparent competition, community ecology, facilitation, indirect effects, inducible defences, microcosms, predator–prey dynamics, structural equation modelling

## Abstract

1. Apparent competition is one mechanism that can contribute to the complex dynamics observed in natural systems, yet it remains understudied in empirical systems. Understanding the dynamics that shape the outcome of processes like apparent competition is vital for appreciating how they influence natural systems.
2. We empirically evaluated the role of indirect trophic interactions in driving apparent competition in a model laboratory system. Our experimental system was designed to let us evaluate combined direct and indirect interactions among species.
3. Here we describe the results of a factorial experiment using two noncompeting prey (*Colpidium kleini*, a heterotroph, and *Chlamydomonas reinhardtii*, an autotroph) consumed by a generalist predator *Euplotes eurystomus* to explore the dynamics of apparent competition. To gain intuition into the potential mechanism driving the asymmetry in the observed results, we further explored the system using structural equation modelling.
4. Our results show an important role of positive interactions and indirect effects contributing to apparent competition in this system with a marked asymmetrical outcome favouring one prey, *Chlamydomonas*. The selected structural equation supports a role of indirect facilitation; although *Chlamydomonas* (a photoautotroph) and *Colpidium* (a bacterivore) use different resources and therefor do not directly compete, *Colpidium* reduces bacteria that may compete with *Chlamydomonas*. In addition, formation of colonies by *Chlamydomonas* in response to predation by *Euplotes* provides an antipredator defence not available to *Colpidium*.
5. Asymmetric apparent competition may be more common in natural systems than the symmetric interaction originally proposed in classic theory, suggesting that exploration of the mechanisms driving the asymmetry of the interaction can be a fruitful area of further research to better our understanding of interspecific interactions and community dynamics.

## Graphical abstract

This work explores the complex influences that can shape the outcomes of apparent competition.

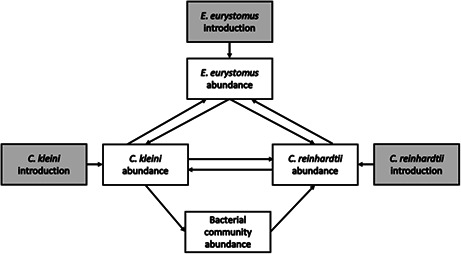

## 1 INTRODUCTION

Apparent competition, an indirect interaction in which the presence of two or more non‐competing prey increases shared predator abundance thereby reducing prey abundances, has been explored as a means of understanding complex ecological outcomes (Holt & Bonsall, 2017; Holt & Lawton, 1994).The integration of apparent competition (Holt, 1977) into studies of broader ecological frameworks is critical to understanding how direct and indirect effects interact in theory (e.g. Caudera et al., 2021; Seno et al., 2020; Stige et al., 2018) and in natural ecological communities (Anderson et al., 2018; Holt & Bonsall, 2017; Holt & Lawton, 1994). Apparent competition can influence community structure and functioning in a variety of ways (Frost et al., 2016; Morris et al., 2004) such as promoting the stable coexistence of prey species (Grover & Holt, 1998; Tilman, 2007) or exclusion of community members (Banerji & Morin, 2014; Bonsall & Hassell, 1997; Holt & Bonsall, 2017; McPeek, 2019). Although research has demonstrated that many natural systems display aspects of apparent competition (DeCesare et al., 2009; Dunn et al., 2012; Holt & Lawton, 1994; Neufeld et al., 2021), the complex combination of direct and indirect interactions can often restrict the ability to determine the mechanisms governing the type of symmetry seen between prey as a result of apparent competition.

Despite the difficulty of observing indirect interactions (Orrock et al., 2015; Wootton, 1994), indirect effects may be as important as direct effects in influencing community dynamics (Bonsall & Hassell, 1997; Han et al., 2020). More recent theoretical and subsequent empirical work by Stige et al. (2018, 2019) indicates that although it can be difficult to infer and interpret indirect effects, evaluating top‐down and bottom‐up effects in these systems can lead to greater understanding of the role of apparent competition for food web dynamics. This is especially likely for apparent competition given the number of factors that can promote, modify, or eliminate its effects (Tack et al., 2011). A major prediction of a number of models (e.g. Holt et al., 1994) is that one prey will be an inferior apparent competitor and such cases of asymmetry appear to be common in nature (Chaneton & Bonsall, 2000). One mechanism that might play a role in shaping asymmetric outcomes of apparent competition is a change in prey suitability (e.g. Holt & Kotler, 1987). Specifically, if prey suitability declines (e.g. due to an induced defence) predator attack rates or assimilation efficiency might decline, altering the outcome for the prey beyond what would be suggested in early theoretical models, potentially exacerbating or even driving such an observed asymmetry. These studies highlight the need to understand how combinations of processes, such as bottom‐up effects and apparent competition, can modulate community‐level changes.

Understanding positive effects in ecology is a needed extension of niche theory and community ecology to better explain complex dynamics (Koffel et al., 2021). The roles of indirect positive effects, such as facilitation, are being incorporated into broader ecological concepts with increasing frequency (Bruno et al., 2003; Bulleri et al., 2016; Michalet & Pugnaire, 2016; Stachowicz, 2001; Wright et al., 2017), including apparent competition (Allesina & Levine, 2011). A combination of direct and indirect facilitation has been observed, although the strength of facilitation appears to be highly context‐dependent (Cuesta et al., 2010; Michalet et al., 2015). Conceptual models predict that interspecific prey facilitation should increase prey abundance (Bruno & Bertness, 2001), leading to increased predator abundance (Bulleri et al., 2016). There is also support for the role of mutualisms in systems experiencing apparent competition (Abrams et al., 1998; Costa & Anjos, 2020; Long et al., 2012), although such effects are contingent on the relative densities of the prey. These conceptual frameworks, coupled with the observed role of facilitation in regulating community structure (Butterfield, 2009; Butterfield & Callaway, 2013; Lortie et al., 2021) and biodiversity (Bulleri et al., 2018; McIntire & Fajardo, 2009) make establishing the link between facilitation and apparent competition crucial for understanding outcomes of these indirect effects.

Here we describe a factorial experiment that revealed the role of facilitation by prey species in shaping outcomes of apparent competition on predator and prey abundances in a community of protists. Two prey species, the heterotrophic ciliate *Colpidium kleini* and the autotrophic green alga *Chlamydomonas reinhardtii*, are unlikely to compete directly given their different trophic positions (bacterivore and photoautotroph, respectively), and both prey species are consumed by the ciliated predator *Euplotes eurystomus*. Aside from apparent competition, two additional factors had the potential to complicate the dynamics we observed between our three study species. First: the inclusion of a standardized bacterial community as prey for *Colpidium* also presented a potential competitive challenge to the alga *Chlamydomonas*. Algae and bacteria frequently compete for nutrients such as phosphorus (Grover, 2000; Løvdal et al., 2007), and high turbidity caused by abundant bacteria could also potentially depress algal growth by reducing the light available for photosynthesis (Wang, 1974). Second: *Chlamydomonas* responds to predators by forming non‐motile, multicellular colonies that that reduce predation by small filter feeding micrograzers (e.g. Becks et al., 2010; Lurling & Beekman, 2006) and create a refuge from elevated predator abundances resulting from apparent competition. These colonies are advantageous to *Chlamydomonas* populations experiencing predation but come with a trade‐off in restricting the colonies to a suboptimal region of their environment in terms of access to light and nutrients. Although these colonies provide a partial defence against predators like *Euplotes*, the defence is incomplete at the population level (both unicells and colonies of *Chlamydomonas* persist in culture under predation) and the colonial subpopulation may continue to contribute unicellular *Chlamydomonas* over time as predator abundances fluctuate.

We used a series of factorial treatments containing subsets of our three protist species to address three specific questions. (1) Do the prey *Chlamydomonas* and *Colpidium* experience apparent competition when interacting with the generalist predator *Euplotes*? (2) Does consumption of the bacterial community by *Colpidium* facilitate the alternate prey *Chlamydomonas?* and (3) Does the formation of defensive colonies by *Chlamydomonas* result in that species being a superior apparent competitor? We expected that the two prey would exhibit apparent completion and that the formation of defensive colonies by *Chlamydomonas* and facilitation of *Chlamydomonas* by *Colpidium* would result in an asymmetric outcome favouring the alga.

## 2 MATERIALS AND METHODS

### 2.1 Organisms and culturing conditions

To evaluate the mechanisms and effects of apparent competition in our model laboratory community, we established treatments with three species in a factorial design. The alga *C. reinhardtii* (a photoautotroph) and the ciliated protist *C. kleini* (a bacterivorous heterotroph) are unlikely to compete directly, and each has the ability to support populations of our chosen ciliated predator, *E. eurystomus*. The ability of both prey species to support the growth of *Euplotes* and observations in feeding trials that *Colpidium* did not consume significant numbers of *Chlamydomonas* suggested these species would be suitable for evaluating the potential for apparent competition. Cultures of *Chlamydomonas* (CC‐1010) originated from the *Chlamydomonas* Resource Center (University of Minnesota). Populations of *Euplotes* and *Colpidium* were originally obtained from Carolina Biological Supply Company (Burlington, NC) and the Adelphia Plant Science Research and Extension Center (Freehold, New Jersey) respectively. Although the protist populations used in this study were obtained separately, members of these genera co‐occur in freshwater ecosystems in the northeastern United States and are likely representative of the interactions between similar taxa. Approval for the ethical treatment of animals was not required given that this experiment only used unicellular eukaryotes and prokaryotes.

Monospecific stock cultures of the three experimental species grew in microcosms (i.e. autoclave‐sterilized, loosely lidded 250 ml glass jars as used in Banerji & Morin, 2014; Faillace & Morin, 2016). Although *Euplotes* require other smaller protists as prey, their populations will persist without growth for weeks with a suitable bacterial community. Microcosms contained 100 ml of autoclave‐sterilized complex organic medium made with 0.4 grams of Carolina Biological Supply protozoan pellets (Carolina Biological Supply Company) and 0.14 g Herptivite nutrient supplement to 1 L of filtered well water collected from Somerset, New Jersey. Well water was filtered through Whatman filters to remove particulate matter before sterilization. Sterile medium received an inoculum of four bacterial taxa (*Serratia marcescens, Bacillus subtilis, Bacillus cereus* and *Proteus vulgaris*) to standardize the bacterial community composition across treatments before introduction of protists. *Chlamydomonas* was initially cultured using a 1:1 mixture of TAP medium (Gorman & Levine, 1965) and the previously described organic medium. Our choice of media was intended to prevent nutrient limitation and minimize any nutrient‐related competitive effects in our study.

### 2.2 Experimental set‐up

We created seven treatments containing all possible combinations of the three experimental species: (*Euplotes* alone, *Chlamydomonas* alone, *Colpidium* alone, *Colpidium* and *Euplotes*, *Chlamydomonas* and *Euplotes*, *Colpidium* and *Chlamydomonas*, *Euplotes* with *Colpidium* and *Chlamydomonas*) and an additional eighth treatment consisted of a protist‐free control used to monitor bacterial abundance in the absence of protists (*n* = 5 for each treatment). Positions of microcosms were randomized in a Percival incubator at 24°C with a 12‐hr light: 12‐hr dark photoperiod. All experimental microcosms contained two sterile wheat seeds for additional nutrients. An addition of 50 ml of bacterized medium to 50 ml of dense algal culture (>1 × 10^6^ cells/ml) initiated any treatments containing *Chlamydomonas*. We introduced *Colpidium* and *Euplotes* by transferring 20 individuals of each species from stock cultures using micropipettes. Prey grew for an initial period of 9 days without predators to ensure establishment of *Chlamydomonas* and *Colpidium*. *Euplotes* were added to appropriate treatments on day 10 and grew for a subsequent week of additional population growth before sampling to ensure that predators had sufficient time to become established. This experimental design therefore evaluated a minimum of approximately 70 protozoan prey generations and 40 predator generations. We monitored microcosms weekly to measure pH, to adjust volume due to evaporation and to evaluate them for bacterial or fungal contamination.

Every 2 days for 2 weeks (eight sample points total) after the introduction of predators we measured the abundances of all three species by removing a 1 ml subsample from well‐mixed microcosms and estimated abundances of each species. Data consisted of counts of *Colpidium* and *Euplotes* using a Nikon SMZ microscope at 20X magnification and counts of unicellular *Chlamydomonas* using a Reichert haemocytometer and a Nikon Eclipse 80i compound microscope at 400X magnification with phase contrast.

We sampled both *Chlamydomonas* colonies (which form when grazed by *Euplotes*) and turbidity of homogenized growth medium (to measure bacterial abundance) in appropriate treatments at the end of our 3‐week experimental period. We collected subsamples and measured the abundance of *Chlamydomonas* colonies in the two treatments that contained both *Chlamydomonas* and *Euplotes* as described previously for single cells. Colony data were not collected in any other treatments unless they became detectable. Turbidity provided an approximation of the relative concentrations of the bacterial communities (Monod, 1949). For each replicate, optical density at 590 nm (selected to reduce influence of *Chlamydomonas* on turbidity) of gently shaken growth medium estimated the approximate abundances of bacteria in each treatment (using a Milton‐Roy 601 spectrophotometer). To similarly evaluate a protist‐free control, we measured optical density of our treatment containing only bacteria that was initiated after the original data collection but was cultured for an equal time period under the same culturing conditions.

### 2.3 Statistical analyses

We calculated mean density per mL for *Chlamydomonas* unicells, *Euplotes*, and *Colpidium* averaged over time points after the establishment of *Euplotes* (*n* = 6). Means averaged over time were then log_10_‐transformed and analysed with analyses of variance (ANOVA) for each species. Tukey's honestly significant difference tests for multiple comparisons evaluated significant differences in treatment means for each species at the 0.05 level of significance. We performed an ANOVA for turbidity data across all treatments and a t‐test for log_10_‐transformed abundances of *Chlamydomonas* colonies in the two treatments where colonies were detectable for the final time point in the sampling period. ANOVA and t‐tests were performed using SAS (SAS Institute, 2011 v9.4).

**FIGURE 1:**
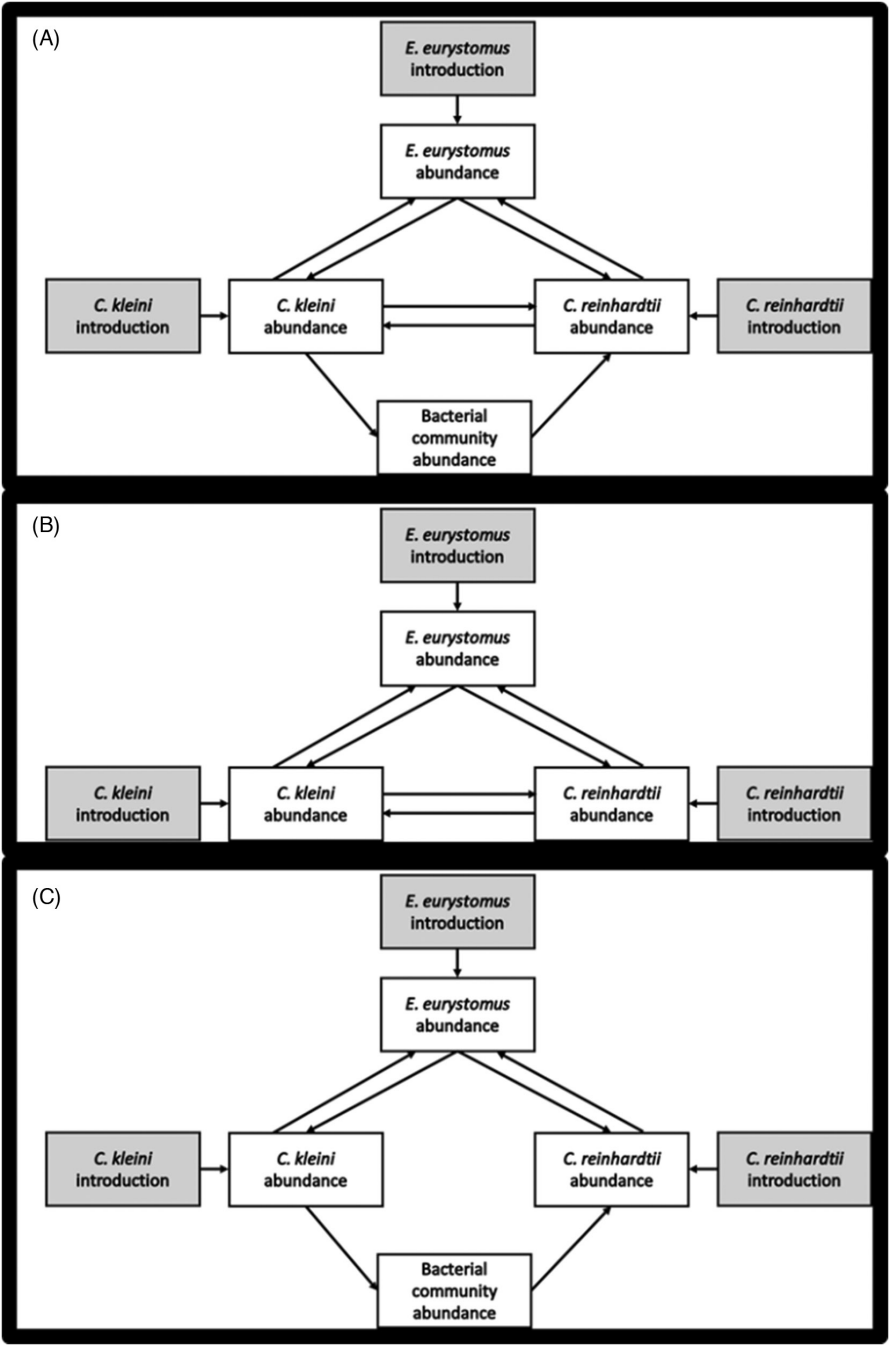
Candidate structural equation models used to examine the possibility of facilitation between the two prey species, *Colpidium kleini* and *Chlamydomonas reinhardtii*. The full model (A), contains all possible paths in our system), the competition model (B) contains a direct feedback between the two prey species and indirect mediation through the abundance of the predator, *Euplotes eurystomus*, but no mediation through the bacterial community, and the mediation model (C) contains only indirection interactions between the prey species mediated through the abundances of the predator and the bacterial community.

We constructed structural equation models (SEMs) in an effort to understand the potential mechanism driving the unexpected results from the ANOVAs, in particular, the possibility of facilitation of *Chlamydomonas* by *Colpidium*. Model construction, fit testing, and analyses were performed using the lavaan package (Rosseel, 2012, version 0.6–7) in R (R Core Team, 2018, version 4.2.0). To incorporate feedbacks between the predator and prey species, as well as potentially between the prey species, we used exogenous ‘introduction variables’ associated singly with each target species that corresponded to the treatment introductions. These introduction variables thus provide unique information for each species in the feedback so that are ‘statistically anchored’ (Kline, 2011). Feedbacks otherwise result in a non‐recursive model structure that is unidentified, making it impossible to evaluate our food web using SEM—the introduction variables thus allowed us to evaluate a single model containing both bottom‐up and top‐down effects simultaneously. We were unable to include *Chlamydomonas* colonies within the model because *Chlamydomonas* colonies form in the presence of *Euplotes*, the predator, and were not intentionally introduced. Thus, without a separate exogenous introduction variable, we were unable to evaluate their feedback with *Chlamydomonas* unicells and excluded them from the model. We assessed model fit using a chi‐square test for three candidate models: a full model with all possible relevant paths among endogenous response variables (i.e. species' abundances; Figure 1A), a second competition model with both predator–prey interactions and direct interactions between the two prey species (*Colpidium* and *Chlamydomonas*; Figure 1B), and a last model that contained, in addition to predator–prey interactions, a uni‐directional effect of *Colpidium* on *Chlamydomonas* mediated through the bacterial community (Figure 1C).

We note that the results from the SEMs should be treated cautiously, as our sample size was quite small (*N* = 35). Although five samples per estimated path is generally recommended as a minimum, here we had 3.2 samples per estimated path for the full model and 3.9 samples per estimated path each for the competition and mediation models. Nonetheless, we believe that the nature of our data (i.e. that they come from a highly controlled, manipulative experiment in contrast to the more common observational data typically analysed with SEMs) allows us to make useful inference from the SEMs despite small sample size.

## 3 RESULTS

### 3.1 Apparent competition between *Chlamydomonas* and *Colpidium*

**FIGURE 2:**
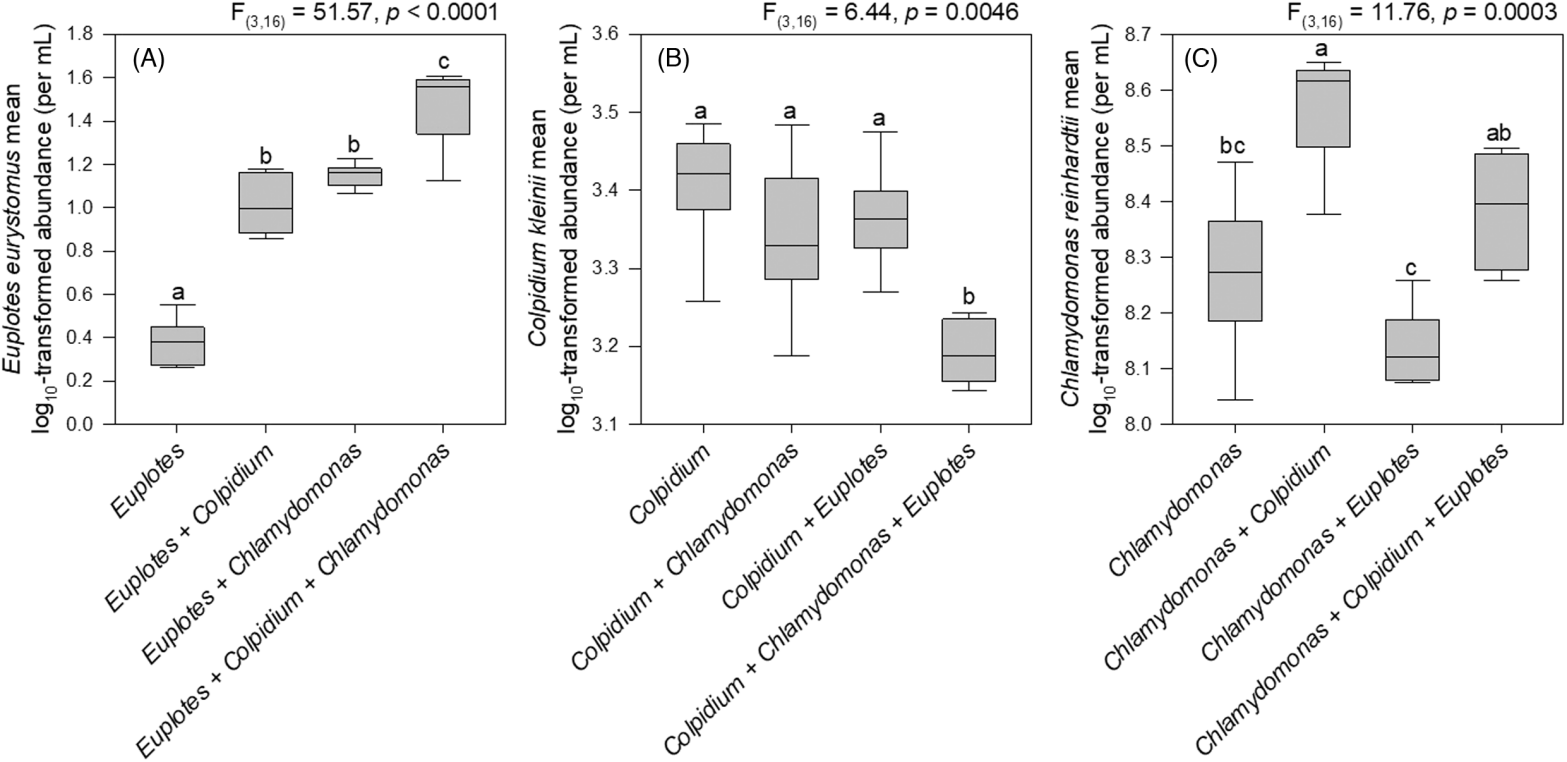
Mean log_10_‐transformed abundances for *Euplotes eurystomus* (A), *Colpidium kleini* (B), and *Chlamydomonas reinhardtii* unicells (C). Letters above boxes indicate treatments that group significantly in Tukey's honestly significant difference. Box plots: Middle line, median; box, interquartile range; whiskers, 5th and 95th percentiles.

When the predator *Euplotes* fed on both *Chlamydomonas* and *Colpidium* together the mean abundance for the predator more than doubled compared with when it was cultured with either prey species separately, and *Euplotes* density was more than 10 times higher than when cultured on bacteria alone (*F*_3,16_ = 51.57, *p* < 0.0001, Figure 2A). The increase in predator abundance when feeding on both *Chlamydomonas* and *Colpidium* was accompanied by a significant decrease in the abundance of *Colpidium* relative to the treatment where *Euplotes* fed only on *Colpidium*. *Colpidium* significantly declined in abundance relative to predator‐free controls only when *Chlamydomonas* was also present (*F*_3,16_ = 6.44, *p* = 0.0046, Figure 2B). Although different treatments did affect *Chlamydomonas* unicell abundances (*F*_3,16_ = 11.76, *p* = 0.0003), unlike the pattern displayed by *Colpidium*, the abundance of unicellular *Chlamydomonas* coexisting with *Euplotes* actually more than doubled when *Colpidium* was present (Figure 2C), relative to controls.

### 3.2 Influence of *Colpidium* on bacterial abundance

**FIGURE 3:**
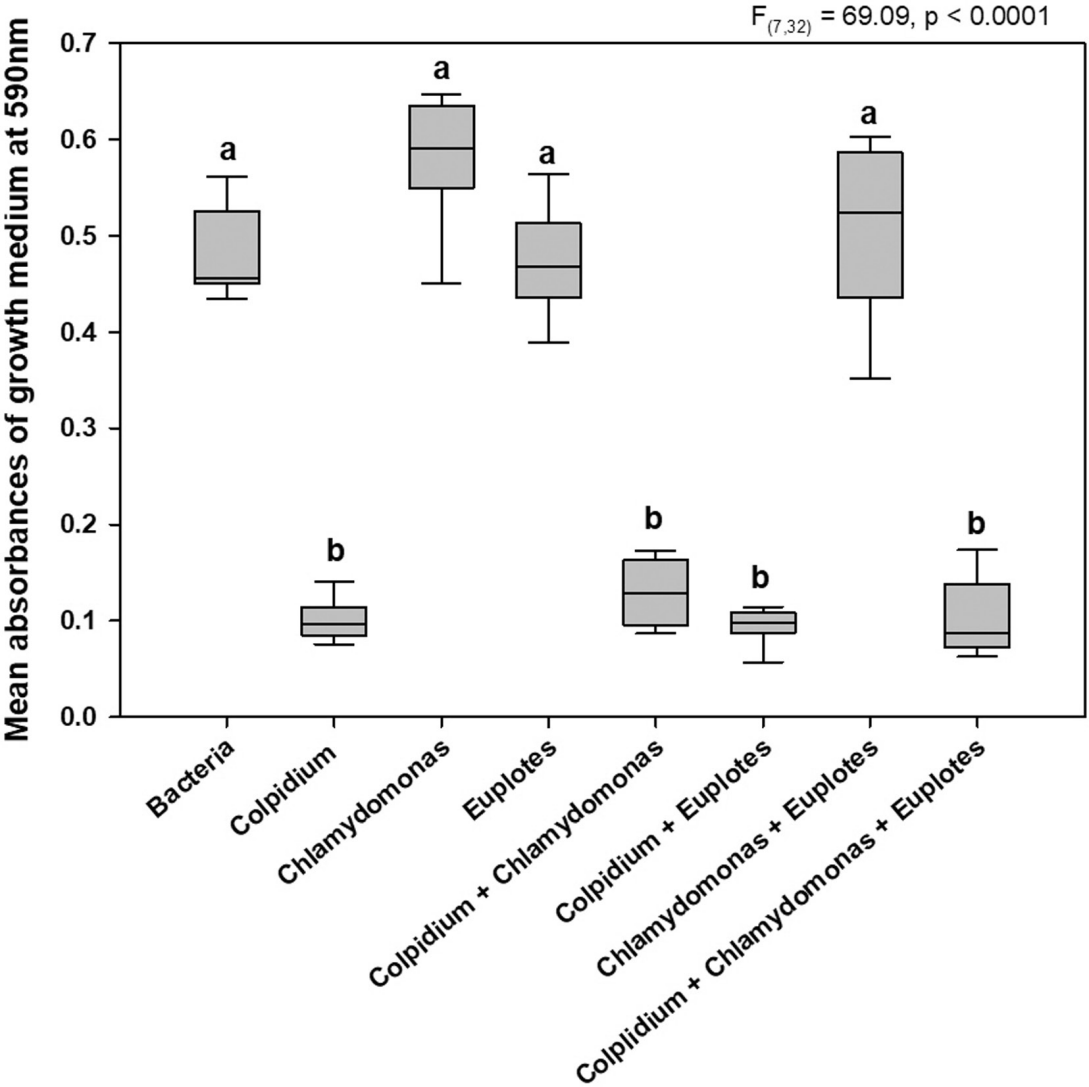
Absorbance data of all experimental treatments measured at 590 nm at the end of experiment as a measure of medium turbidity. Reduced absorbance implies reduced bacterial abundance. Letters above boxes indicate treatments that group significantly in Tukey's honestly significant difference. Box plots: Middle line, median; box, interquartile range; whiskers, 5th and 95th percentiles.

The positive effect of *Colpidium* on *Chlamydomonas* abundance, observed with or without predation by *Euplotes*, is associated with the depression of bacterial abundance as assessed by relative turbidity across treatments. All treatments containing *Colpidium* displayed similarly low levels of turbidity (*F*_7,32_ = 69.09, *p* < 0.0001), which are consistent with lower bacterial abundance. All treatments without *Colpidium* had higher turbidity and were indistinguishable from a comparison treatment containing only bacteria without protists (Figure 3). In treatments where *Chlamydomonas* and *Colpidium* were both present algal abundances were significantly higher in concert with reduced bacterial abundance.

### 3.3 Induction of *Chlamydomonas* defensive colonies by *Euplotes*

Unicellular *Chlamydomonas* formed multicellular colonies in communities containing the predator *Euplotes*. Colonies did not appear at detectable levels when *Chlamydomonas* grew without *Euplotes* (i.e. in *Chlamydomonas* controls or with only *Colpidium*). There was a slight but statistically significant increase (*t*_6_ = −2.4109, *p* = 0.042) in *Chlamydomonas* colonies in the treatment containing *Euplotes* and *Colpidium* compared with cultures containing only *Chlamydomonas* and *Euplotes* (Figure S1). Although the predators triggering colony formation doubled between these two experimental treatments containing colonies, the number of colonies themselves only sees a 41% increase.

### 3.4 Structural equation model fit and results

Chi‐square tests for all three models indicated poor global fit (Table 1). Inspection of the model and sample covariance matrices indicated that global model fit was likely affected by the inclusion of the exogenous introduction variables whose inclusion was necessary to evaluate feedbacks in the model (see Section 2). Specifically, the model design requires that each introduction variable only be linked to its associated endogenous response variable (species' abundance), leaving multiple unspecified paths between exogenous introduction variables to additional endogenous variables. For example, only the path from ‘*C. kleini* introduction’ to ‘*C. kleini* abundance’ is specified in our models, leaving unspecified potential paths from ‘*C. kleini* introduction’ to ‘*E. eurystomus* abundance’, ‘*C. reinhardtii* abundance’ and ‘bacterial community abundance’. With the preceding factors in mind, we used Akaike's information criterion adjusted for small sample size (AICc) to compare our three candidate models. To ensure comparable model structure for the competition model relative to the full and mediation models, the paths between *Colpidium* and the bacterial community and between the bacterial community and *Chlamydomonas* were constrained (i.e. set to zero), but the bacterial abundance variable remained in the model.

**TABLE 1:** Global fit statistics for three candidate models (*N* = 35 for each). We report Akaike's information criterion corrected for small sample size. The selected model based on AICc was the mediation model (in bold font). Significance is denoted using ‘*’ for *p* values less than 0.005, ‘**’ for *p*‐values less than 0.002 than 0.001 and *** for any value equal or less than 0.001

Model	Chi‐square	*df*	*p*‐value	CFI	TLI	RMSEA	SRMR	AICc
Full	22.73	7	0.002 (**)	0.976	0.938	0.253	0.032	−205.6
Competition	110.33	9	0.000 (***)	0.884	0.689	0.567	0.265	−129.9
**Mediation**	**29.61**	**9**	0.001 (***)	**0.968**	**0.937**	**0.256**	**0.036**	**−210.7**

**FIGURE 4:**
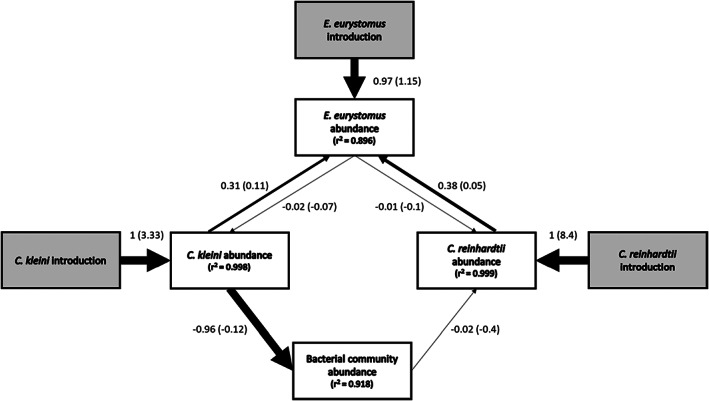
Path diagram showing the relationships among species interacting in our community for the selected mediation model. *Euplotes eurystomus* is the predator, while *Colpidium kleini* and *Chlamydomonas reinhardtii* are the two prey species. Grey boxes indicate the exogenous introduction variables used to enable us to evaluate the feedbacks within the model. All paths in the model were significant (*α* = 0.01). Path widths are proportional to the standardized coefficients shown next to each significant path. Unstandardized coefficients are shown in parentheses for each path. *R* ^2^ values (1 – Ratio of the residual variance) for each endogenous variable are shown in the boxes.

Although global model fit was poor for all models, *R* ^2^ values for all endogenous variables were large (Figure 4). These large *R* ^2^ values support our assertion that meaningless paths in the model are biasing global fit (Lefcheck et al., 2018). For this reason, we proceeded with model selection and analysis of results despite poor global fit (Table 1). We selected the mediation model as the most parsimonious model after examination of the AICc scores (Table 1). Further examination of the mediation model suggests that all interactions among species are significant (Figure 4, Table 2).

**TABLE 2:** Regression coefficients for all individual paths within the selected mediation model. All values are log_10_‐transformed. All paths are significant. Significance is denoted using ‘*’ for *p* values less than 0.005, ‘**’ for *p*‐values less than 0.002 than 0.001 and *** for any value equal or less than 0.001

Response	Predictor	Estimate	*SE*	*z*‐value	*p*‐value	Standardized coefficient
*Euplotes Eurystomus*	*E. eurystomus* introduction	1.146	0.067	17.087	0.000 (***)	0.971
*E. eurystomus*	*Colpidium kleini*	0.111	0.020	5.542	0.000 (***)	0.313
*E. eurystomus*	*Chlamydomonas reinhardtii*	0.053	0.008	6.654	0.000 (***)	0.376
*C. kleini*	*C. kleini* introduction	3.334	0.025	133.307	0.000 (***)	1.001
*C. kleini*	*E. eurystomus*	−0.067	0.021	−3.149	0.002 (**)	−0.024
*C. reinhardtii*	*C. reinhardtii* introduction	8.399	0.039	217.014	0.000 (***)	1.005
*C. reinhardtii*	*E. eurystomus*	−0.096	0.032	−2.949	0.003 (**)	−0.014
*C. reinhardtii*	Bacterial abundance	−0.396	0.089	−4.457	0.000 (***)	−0.020
*C. reinhardtii*	Indirect effect of *C. kleini*	0.020	0.005	4.347	0.000 (***)	0.028
Bacterial abundance	*C. kleini*	−0.124	0.006	−19.796	0.000 (***)	−0.958

In agreement with the ANOVA results, both prey species positively influenced the abundance of the predator, which in turn negatively influenced the abundance of the two prey species. *Colpidium* depressed the abundance of bacteria, which itself had a negative impact on the abundance of *Chlamydomonas*. Based on our selected model, the SEM confirms that *Colpidium* has an indirect positive impact on the abundance of *Chlamydomonas* mediated through the bacteria. It should be noted that the unusually high *R* ^2^ values for the variables in our models are unsurprising in our system as they derive from the inclusion of the exogenous introduction variables with which the respective species' abundances are inevitably highly correlated.

## 4 DISCUSSION

As predicted, when *C. kleini* and *C. reinhardtii* occurred together with the predator *E. eurystomus*, we observed depressed prey abundance and increased predator abundance consistent with apparent competition. We also observed an asymmetric positive effect of *Colpidium* on *Chlamydomonas* that we attribute to a reduction in bacteria that inhibited algal growth when these two species grew together, regardless of whether the predator was present or absent (Figure 2C). The asymmetry observed (Figure 4) is consistent with other commonly observed asymmetrical outcomes between prey in systems displaying apparent competition (Chaneton & Bonsall, 2000). The results of our path analysis also lend support to our second prediction that factors unrelated to the apparent competition (including facilitation) strongly influenced the outcome. The interplay of direct and indirect effects in our model communities creates a fascinating chain reaction in which (1) *Colpidium* reduces bacterial abundances and consequently promotes *Chlamydomonas* abundance, (2) The combined presence of *Colpidium* and *Chlamydomonas* increases *Euplotes* abundance and results in apparent competition. Only the facilitating prey (*Colpidium*), however, seems to suffer the consequences, perhaps because (3) The formation of *Chlamydomonas* colonies scales with increased predator abundance and potentially facilitates persistence of the *Euplotes* and *Chlamydomonas* interaction as explored in previous theoretical work (e.g. Grover & Holt, 1998), perpetuating the apparent competition and resulting in the asymmetrical pattern we observed.

There is a need to relate food web dynamics and processes to the various outcomes of apparent competition (Holt & Bonsall, 2017) to better understand how these outcomes arise. Our results allow us to integrate previous work on predation, apparent competition and the role of inducible defences in community dynamics. Models of species coexistence support the idea that predator–prey interactions can be stabilizing (Allesina & Tang, 2012; Brose et al., 2019), and many examples of top‐down drivers for maintaining diversity have been found in natural systems (Terborgh, 2015). In addition to the more general role of predation for enabling species coexistence, anti‐predator defensive phenotypes can also be important in driving persistence of focal groups (e.g. Aránguiz‐Acuña et al., 2010) or communities as a whole (e.g. Boeing & Ramcharan, 2010). A number of factors could cause the asymmetrical outcome seen in our results, but we suggest the formation of defensive colonies by *Chlamydomonas* and the turbidity reduction caused by *Colpidium* as being the most significant drivers in this system. The role of *Chlamydomonas* defensive colonies in our experimental system provides a case like that predicted by Grover and Holt (1998), in which coexistence results from one prey being better defended against predation (*Chlamydomonas* forming colonies which favour defence over population growth rate) and one prey specializing on resource acquisition (*Colpidium*). Although the *Chlamydomonas* defensive phenotype does likely provide an advantage, our empirical results suggest that the asymmetry in prey abundance is more likely due to another cause – the facilitation of *Chlamydomonas* by *Colpidium*.

Theory that has considered differences in ways predators obtain prey in multi‐prey systems would suggest that attack rate might decline as predator abundance increases (e.g. Holt and Kotler 1987) in cases like those seen in our experiment (where colony formation increases as a function of predator abundance and lowers attack rates). Although we believe colonies do provide a defence against predation, that defence is incomplete at the population level (both colonies and single celled *Chlamydomonas* phenotypes persist under predation). It remains unclear whether single cells can reenter the water column from the colonial phenotype, but our data suggest an alternate source of increased unicellular algal abundance in the form of *Colpidium‐*mediated bacterial suppression. Single cells of *Chlamydomonas* significantly increase in abundance even in the absence of predation (and corresponding colony formation) so long as *Colpidium* is also present (Figure 2C). Furthermore, *Chlamydomonas* cultured with *Euplotes* in unbacterized medium also show significantly elevated abundance of unicells despite elevated predation and resulting colony formation in those conditions (NSL, unpublished data). These results suggest that interactions between the prey species in isolation of the predator might have a stronger influence on observed algal unicell abundances compared with the colony formation that occurs in the presence of the predator.

The fact that *Chlamydomonas* unicell abundances were unaffected by elevated predator abundances resulting from apparent competition was surprising compared with more traditional systems experiencing apparent competition where prey would be both experience depressed abundances. Given that *Chlamydomonas* and *Colpidium* are equally capable of supporting *Euplotes* populations when either prey occurs alone, and *Chlamydomonas* abundances are significantly higher in the presence of *Colpidium* one might anticipate depressed abundances of *Chlamydomonas* under apparent competition. The results of our selected mediation model (Figure 4) supports our interpretation that the interaction between the predator *Euplotes* and the combined prey species results in apparent competition that is asymmetrical due to dynamics between *Chlamydomonas* and *Colpidium*.

Our SEMs suggested that our data most closely match the apparent competition model, with interactions between the prey, *Colpidium* and *Chlamydomonas*, solely mediated through the abundance of the predator, *Euplotes*, and the bacteria. The apparent competition model has stronger support than either the full model with direct interactions between the prey and the indirectly mediated effects, or the model lacking an interaction mediated through the bacterial community. The selected model confirms that both prey increase the abundance of the predator, with their own abundances concurrently reduced by the predator. The model indicates an additional significant indirect path between *Colpidium* and *Chlamydomonas* mediated through the bacteria. As a bacterivore, *Colpidium* has a very strong negative effect on the abundance of bacterial taxa (turbidity in our study being a proxy for bacterial abundance). Increased turbidity has a smaller, but still significant negative effect on *Chlamydomonas*, such that the indirect effect of *Colpidium* on *Chlamydomonas* is positive and nearly twice as strong as that of the predator on *Chlamydomonas* (standardized coefficient of 0.028 for the indirect effect of *Colpidium* versus −0.018 for the effect of *Euplotes*). Although we did not directly evaluate interactions between *Chlamydomonas* and bacteria in our system, we suggest that the algal population was not influenced by competitive interactions (e.g. nutrient limitation) and instead was more strongly influenced by the effect of the relative bacterial abundance on turbidity (Figure 3). Given that the experimental microcosms were designed to avoid nutrient limitation, the changes in prey and predator abundances observed are most likely due to interactions between members of the experimental community. A reduction in turbidity would provide more light to *Chlamydomonas*, enabling it increase its photosynthesis while increased turbidity would have the inverse effect. The results of our models provide additional support that the asymmetrical nature of the apparent competition we observed is the result of indirect facilitation between the two prey species. Coupled with the defensive phenotype shown by part of the *Chlamydomonas* population – this would create conditions where persisting *Chlamydomonas* single cells would still bolster *Euplotes* abundance and serve as a consistent pressure in promoting the asymmetry observed.

Although cases of predator facilitation of prey species have been considered previously (e.g. Pope et al., 2008), our results highlight a need to explore cases where facilitation between the prey themselves can influence apparent competition in ways that affect the fitness consequences for both prey species (Schöb et al., 2014). Indirect facilitation like that observed in our system has been linked to negative interactions previously (Adams et al., 2003; Flory & Bauer, 2014; Wright et al., 2017), but the roles of such indirect effects are far from clear (Cuesta et al., 2010; Soliveres et al., 2015). By considering indirect facilitation, we can broaden our understanding of ecological interactions in complex systems (Cavieres, 2021; Saccone et al., 2010). Our findings support previous evidence that facilitation can modify the strength of negative interactions between species (Bulleri et al., 2016; Li et al., 2019) and contribute to changes in species abundances by modifying interactions at the community level (Levine, 1999). Because facilitation and apparent competition contribute to shared processes at the community level (e.g. modifying interaction strength, community structure, invasions and conservation, etc.), improving our understanding of the interplay between the positive effect of facilitation and the negative effect of apparent competition should be a focus of future work.

Our study explored the role of mixed direct and indirect effects; including facilitation and trait‐mediated indirect effects, in shaping the outcomes of apparent competition. This raises a number of interesting questions for fields such as invasion and conservation biology, where both apparent competition and facilitation have been observed to play a pronounced role (Bhattarai et al., 2017; Mumma et al., 2018; Strauss et al., 2012; Wittmer et al., 2013). More work is required to disentangle the combination of direct and indirect effects that ultimately combine to shape the outcomes observed from apparent competition and how effects may be promoted or diffused in natural systems. By exploring how these interactions are shaped and modified we hope to expand our understanding of the role of positive interactions in governing the outcomes of apparent competition.

## AUTHOR CONTRIBUTIONS

N.S.L. and C.A.F. designed the conceptual framework for the study; N.S.L. designed the community used, collected all data and analysed turbidity and *Chlamydomonas* colony data; C.A.F. conducted all other statistical analyses; N.S.L. and C.A.F. jointly wrote the manuscript.

## CONFLICT OF INTEREST

The authors claim no conflicts of interest. The authors declare no competing financial interests.

## Supporting information


Figure S1

## Data Availability

All data, R code and SAS code used in the analyses of the reported work are available on Zenodo, https://doi.org/10.5281/zenodo.6615024 (Faillace & Lorusso, 2022).

## References

[jane13768-bib-0001] Abrams, P. A. , Holt, R. D. , & Roth, J. D. (1998). Apparent competition or apparent mutualism? Shared predation when populations cycle. Ecology, 79, 201–212.

[jane13768-bib-0002] Adams, M. J. , Pearl, C. A. , & Bruce Bury, R. (2003). Indirect facilitation of an anuran invasion by non‐native fishes. Ecology Letters, 6, 343–351.

[jane13768-bib-0003] Allesina, S. , & Levine, J. M. (2011). A competitive network theory of species diversity. Proceedings of the National Academy of Sciences of the United States of America, 108, 5638–5642. 21415368 10.1073/pnas.1014428108 PMC3078357

[jane13768-bib-0004] Allesina, S. , & Tang, S. (2012). Stability criteria for complex ecosystems. Nature, 483, 205–208. 22343894 10.1038/nature10832

[jane13768-bib-0005] Anderson, M. , McLellan, B. N. , & Serrouya, R. (2018). Moose response to high‐elevation forestry: Implications for apparent competition with endangered caribou. The Journal of Wildlife Management, 82, 299–309.

[jane13768-bib-0006] Aránguiz‐Acuña, A. , Ramos‐Jiliberto, R. , Sarma, N. , Sarma, S. S. S. , Bustamante, R. O. , & Toledo, V. (2010). Benefits, costs and reactivity of inducible defences: An experimental test with rotifers. Freshwater Biology, 55, 2114–2122.

[jane13768-bib-0007] Banerji, A. , & Morin, P. J. (2014). Trait‐mediated apparent competition in an intraguild predator–prey system. Oikos, 123, 567–574.

[jane13768-bib-0008] Becks, L. , Ellner, S. P. , Jones, L. E. , & Hairston, N. G., Jr. (2010). Reduction of adaptive genetic diversity radically alters eco‐evolutionary community dynamics. Ecology Letters, 13, 989–997. 20528898 10.1111/j.1461-0248.2010.01490.x

[jane13768-bib-0009] Bhattarai, G. P. , Meyerson, L. A. , & Cronin, J. T. (2017). Geographic variation in apparent competition between native and invasive Phragmites australis. Ecology, 98, 349–358. 27861789 10.1002/ecy.1646

[jane13768-bib-0074] Boeing, W. J. , & Ramcharan, C. W. (2010). Inducible defences are a stabilizing factor for predator and prey populations: A field experiment. Freshwater Biology, 55(11), 2332–2338. 10.1111/j.1365-2427.2010.02446.x

[jane13768-bib-0010] Bonsall, M. B. , & Hassell, M. P. (1997). Apparent competition structures ecological assemblages. Nature, 388, 371–373.

[jane13768-bib-0011] Brose, U. , Archambault, P. , Barnes, A. D. , Bersier, L.‐F. , Boy, T. , Canning‐Clode, J. , Conti, E. , Dias, M. , Digel, C. , Dissanayake, A. , Flores, A. A. V. , Fussmann, K. , Gauzens, B. , Gray, C. , Häussler, J. , Hirt, M. R. , Jacob, U. , Jochum, M. , Kéfi, S. , … Iles, A. C. (2019). Predator traits determine food‐web architecture across ecosystems. Nature Ecology & Evolution, 3(6), 919–927. 31110252 10.1038/s41559-019-0899-x

[jane13768-bib-0012] Bruno, J. , and M. Bertness . (2001). Habitat modification and facilitation in benthic marine communities.

[jane13768-bib-0013] Bruno, J. F. , Stachowicz, J. J. , & Bertness, M. D. (2003). Inclusion of facilitation into ecological theory. Trends in Ecology & Evolution, 18, 119–125.

[jane13768-bib-0014] Bulleri, F. , Bruno, J. F. , Silliman, B. R. , & Stachowicz, J. J. (2016). Facilitation and the niche: Implications for coexistence, range shifts and ecosystem functioning. Functional Ecology, 30, 70–78.

[jane13768-bib-0015] Bulleri, F. , Eriksson, B. K. , Queirós, A. , Airoldi, L. , Arenas, F. , Arvanitidis, C. , Bouma, T. J. , Crowe, T. P. , Davoult, D. , Guizien, K. , Iveša, L. , Jenkins, S. R. , Michalet, R. , Olabarria, C. , Procaccini, G. , Serrão, E. A. , Wahl, M. , & Benedetti‐Cecchi, L. (2018). Harnessing positive species interactions as a tool against climate‐driven loss of coastal biodiversity. PLoS Biology, 16, e2006852. 30180154 10.1371/journal.pbio.2006852 PMC6138402

[jane13768-bib-0016] Butterfield, B. J. (2009). Effects of facilitation on community stability and dynamics: Synthesis and future directions. Journal of Ecology, 97, 1192–1201.

[jane13768-bib-0017] Butterfield, B. J. , & Callaway, R. M. (2013). A functional comparative approach to facilitation and its context dependence. Functional Ecology, 27, 907–917.

[jane13768-bib-0073] Caudera, E. , Viale, S. , Bertolino, S. , Cerri, J. , & Venturino, E. (2021). A mathematical model supporting a hyperpredation effect in the apparent competition between invasive eastern cottontail and native European hare. Bulletin of Mathematical Biology, 83(5). 10.1007/s11538-021-00873-9 PMC8004525 33772654

[jane13768-bib-0018] Cavieres, L. A. (2021). Facilitation and the invasibility of plant communities. Journal of Ecology, 109, 2019–2028.

[jane13768-bib-0019] Chaneton, E. J. , & Bonsall, M. B. (2000). Enemy‐mediated apparent competition: Empirical patterns and the evidence. Oikos, 88, 380–394.

[jane13768-bib-0020] Costa, M. I. S. , & Anjos, L. (2020). The occurrence of apparent competition and apparent mutualism in a modeled greenhouse system with two non‐competing pests and a shared biocontrol agent. Neotropical Entomology, 49, 874–881. 33074444 10.1007/s13744-020-00820-8

[jane13768-bib-0021] Cuesta, B. , Villar‐Salvador, P. , Puértolas, J. , Rey Benayas, J. M. , & Michalet, R. (2010). Facilitation of *Quercus ilex* in Mediterranean shrubland is explained by both direct and indirect interactions mediated by herbs. Journal of Ecology, 98, 687–696.

[jane13768-bib-0022] DeCesare, N. J. , Hebblewhite, M. , Robinson, H. S. , & Musiani, M. (2009). Endangered, apparently: The role of apparent competition in endangered species conservation. Animal Conservation, 13, 353–362.

[jane13768-bib-0023] Dunn, A. M. , Torchin, M. E. , Hatcher, M. J. , Kotanen, P. M. , Blumenthal, D. M. , Byers, J. E. , Coon, C. A. C. , Frankel, V. M. , Holt, R. D. , Hufbauer, R. A. , Kanarek, A. R. , Schierenbeck, K. A. , Wolfe, L. M. , & Perkins, S. E. (2012). Indirect effects of parasites in invasions. Functional Ecology, 26, 1262–1274.

[jane13768-bib-0024] Faillace, C. A. , & Lorusso, N. S. (2022). Data and statistical code for analyses presented in Lorusso and Faillace. 2022. Indirect facilitation between prey promotes asymetric apparent competition. Zenodo. 10.5281/zenodo.6615024 PMC9544837 35765925

[jane13768-bib-0071] Faillace, C. A. , & Morin, P. J. (2016). Evolution alters the consequences of invasions in experimental communities. Nature Ecology & Evolution, 1(1). 10.1038/s41559-016-0013 28812559

[jane13768-bib-0025] Flory, S. L. , & Bauer, J. T. (2014). Experimental evidence for indirect facilitation among invasive plants. Journal of Ecology, 102, 12–18.

[jane13768-bib-0026] Frost, C. M. , Peralta, G. , Rand, T. A. , Didham, R. K. , Varsani, A. , & Tylianakis, J. M. (2016). Apparent competition drives community‐wide parasitism rates and changes in host abundance across ecosystem boundaries. Nature Communications, 7, 12644. 10.1038/ncomms12644 PMC5013663 27577948

[jane13768-bib-0027] Gorman, D. S. , & Levine, R. P. (1965). TAP and tris‐minimal medium recipes. Proceedings of the National Academy of Sciences of the United States of America, 54, 1665–1669. 4379719 10.1073/pnas.54.6.1665 PMC300531

[jane13768-bib-0028] Grover, J. P. (2000). Resource competition and community structure in aquatic micro‐organisms: Experimental studies of algae and bacteria along a gradient of organic carbon to inorganic phosphorus supply. Journal of Plankton Research, 22, 1591–1610.

[jane13768-bib-0029] Grover, J. P. , & Holt, R. D. (1998). Disentangling resource and apparent competition: Realistic models for plant‐herbivore communities. Journal of Theoretical Biology, 191, 353–376.

[jane13768-bib-0030] Han, P. , Becker, C. , Le Bot, J. , Larbat, R. , Lavoir, A. V. , & Desneux, N. (2020). Plant nutrient supply alters the magnitude of indirect interactions between insect herbivores: From foliar chemistry to community dynamics. Journal of Ecology, 108, 1497–1510.

[jane13768-bib-0031] Holt, R. D. (1977). Predation, apparent competition, and the structure of prey communities. Theoretical Population Biology, 12, 197–229. 929457 10.1016/0040-5809(77)90042-9

[jane13768-bib-0032] Holt, R. D. , & Bonsall, M. B. (2017). Apparent Competition. Annual Review of Ecology, Evolution, and Systematics, 48, 447–471.

[jane13768-bib-0033] Holt, R. D. , Grover, J. , & Tilman, D. (1994). Simple rules for interspecific dominance in systems with exploitative and apparent competition. The American Naturalist, 144, 741–771.

[jane13768-bib-0070] Holt, R. D. , & Kotler, B. P. (1987). Short‐term apparent competition. The American Naturalist, 130(3), 412–430. 10.1086/284718

[jane13768-bib-0034] Holt, R. D. , & Lawton, J. H. (1994). The ecological consequences of shared natural enemies. Annual Review of Ecology and Systematics, 25, 495–520.

[jane13768-bib-0035] Kline, R. B. (2011). Principles and practice of structural equation models (3rd ed.). The Gilford Press.

[jane13768-bib-0075] Koffel, T. , Daufresne, T. , & Klausmeier, C. A. (2021). From competition to facilitation and mutualism: A general theory of the niche. Ecological Monographs, 91(3). 10.1002/ecm.1458

[jane13768-bib-0036] Lefcheck, J. S. , Orth, R. J. , Dennison, W. C. , Wilcox, D. J. , Murphy, R. R. , Keisman, J. , Gurbisz, C. , Hannam, M. , Landry, J. B. , Moore, K. A. , Patrick, C. J. , Testa, J. , Weller, D. E. , & Batiuk, R. A. (2018). Long‐term nutrient reductions lead to the unprecedented recovery of a temperate coastal region. Proceedings of the National Academy of Sciences of the United States of America, 115, 3658–3662. 29507225 10.1073/pnas.1715798115 PMC5889635

[jane13768-bib-0037] Levine, J. M. (1999). Indirect facilitation: Evidence and predictions from a riparian community. Ecology, 80, 1762–1769.

[jane13768-bib-0038] Li, M. , Wei, Z. , Wang, J. , Jousset, A. , Friman, V. , Xu, Y. , Shen, Q. , & Pommier, T. (2019). Facilitation promotes invasions in plant‐associated microbial communities. Ecology Letters, 22, 149–158. 30460736 10.1111/ele.13177

[jane13768-bib-0039] Long, W. , Gamelin, E. , Johnson, E. , & Hines, A. (2012). Density‐dependent indirect effects: Apparent mutualism and apparent competition coexist in a two‐prey system. Marine Ecology Progress Series, 456, 139–148.

[jane13768-bib-0040] Lortie, C. , Filazzola, A. , Brown, C. , Lucero, J. , & Zuliani, M. (2021). Facilitation promotes plant invasions and indirect negative interactions. Oikos, 130, 1056–1061.

[jane13768-bib-0041] Løvdal, T. , Tanaka, T. , & Thingstad, T. F. (2007). Algal‐bacterial competition for phosphorus from dissolved DNA, ATP, and orthophosphate in a mesocosm experiment. Limnology and Oceanography, 52, 1407–1419.

[jane13768-bib-0042] Lurling, M. , & Beekman, W. (2006). Palmelloids formation in Chlamydomonas reinhardtii: Defence against rotifer predators? Annales de Limnologie ‐ International Journal of Limnology, 42, 65–72.

[jane13768-bib-0043] McIntire, E. J. B. , & Fajardo, A. (2009). Beyond description: The active and effective way to infer processes from spatial patterns. Ecology, 90, 46–56. 19294912 10.1890/07-2096.1

[jane13768-bib-0044] McPeek, M. A. (2019). Mechanisms influencing the coexistence of multiple consumers and multiple resources: Resource and apparent competition. Ecological Monographs, 89, e01328.

[jane13768-bib-0045] Michalet, R. , Brooker, R. W. , Lortie, C. J. , Maalouf, J. P. , & Pugnaire, F. I. (2015). Disentangling direct and indirect effects of a legume shrub on its understorey community. Oikos, 124, 1251–1262.

[jane13768-bib-0046] Michalet, R. , & Pugnaire, F. I. (2016). Facilitation in communities: Underlying mechanisms, community and ecosystem implications. Functional Ecology, 30, 3–9.

[jane13768-bib-0047] Monod, J. (1949). The growth of bacterial cultures. Annual Review of Microbiology, 3, 371–394.

[jane13768-bib-0048] Morris, R. J. , Lewis, O. T. , & Godfray, H. C. J. (2004). Experimental evidence for apparent competition in a tropical forest food web. Nature, 428, 310–313. 15029194 10.1038/nature02394

[jane13768-bib-0049] Mumma, M. A. , Gillingham, M. P. , Parker, K. L. , Johnson, C. J. , & Watters, M. (2018). Predation risk for boreal woodland caribou in human‐modified landscapes: Evidence of wolf spatial responses independent of apparent competition. Biological Conservation, 228, 215–223.

[jane13768-bib-0050] Neufeld, B. T. , Superbie, C. , Greuel, R. J. , Perry, T. , Tomchuk, P. A. , Fortin, D. , & McLoughlin, P. D. (2021). Disturbance‐mediated apparent competition decouples in a northern boreal Caribou range. The Journal of Wildlife Management, 85, 254–270.

[jane13768-bib-0051] Orrock, J. L. , Dutra, H. P. , Marquis, R. J. , & Barber, N. (2015). Apparent competition and native consumers exacerbate the strong competitive effect of an exotic plant species. Ecology, 96, 1052–1061. 26230025 10.1890/14-0732.1

[jane13768-bib-0052] Pope, K. L. , J. M. Garwood , H. H. Welsh , and S. P. Lawler . (2008). Evidence of indirect impacts of introduced trout on native amphibians via facilitation of a shared predator.

[jane13768-bib-0072] R Core Team . (2018). R: A language and environment for statistical computing. R Foundation for Statistical Computing.

[jane13768-bib-0053] Rosseel, Y. (2012). Lavaan: An *R* package for structural equation modeling. Journal of Statistical Software, 48, 1–36.

[jane13768-bib-0054] Saccone, P. , Pagès, J.‐P. , Girel, J. , Brun, J.‐J. , & Michalet, R. (2010). Acer negundo invasion along a successional gradient: Early direct facilitation by native pioneers and late indirect facilitation by conspecifics. New Phytologist, 187, 831–842. 20487316 10.1111/j.1469-8137.2010.03289.x

[jane13768-bib-0055] SAS Institute . (2011). The SAS system for windows. SAS Institute.

[jane13768-bib-0056] Schöb, C. , Prieto, I. , Armas, C. , & Pugnaire, F. I. (2014). Consequences of facilitation: One plant's benefit is another plant's cost. Functional Ecology, 28, 500–508.

[jane13768-bib-0057] Seno, H. , Schneider, V. P. , & Kimura, T. (2020). How many preys could coexist with a shared predator in the Lotka–Volterra system?: State transition by species deletion/introduction. Journal of Physics A: Mathematical and Theoretical, 53, 415601.

[jane13768-bib-0058] Soliveres, S. , Smit, C. , & Maestre, F. T. (2015). Moving forward on facilitation research: Response to changing environments and effects on the diversity, functioning and evolution of plant communities. Biological Reviews of the Cambridge Philosophical Society, 90, 297–313. 24774563 10.1111/brv.12110 PMC4407973

[jane13768-bib-0059] Stachowicz, J. J. (2001). Mutualism, facilitation, and the structure of ecological CommunitiesPositive interactions play a critical, but underappreciated, role in ecological communities by reducing physical or biotic stresses in existing habitats and by creating new habitats on which many species depend. Bioscience, 51, 235–246.

[jane13768-bib-0060] Stige, L. C. , Eriksen, E. , Dalpadado, P. , & Ono, K. (2019). Direct and indirect effects of sea ice cover on major zooplankton groups and planktivorous fishes in the Barents Sea. ICES Journal of Marine Science, 76, i24–i36.

[jane13768-bib-0061] Stige, L. C. , Kvile, K. Ø. , Bogstad, B. , & Langangen, Ø. (2018). Predator‐prey interactions cause apparent competition between marine zooplankton groups. Ecology, 99, 632–641. 29281755 10.1002/ecy.2126

[jane13768-bib-0062] Strauss, A. , A. White , and M. Boots . (2012). Invading with biological weapons: the importance of disease‐mediated invasions:1249–1261.

[jane13768-bib-0063] Tack, A. J. M. , Gripenberg, S. , & Roslin, T. (2011). Can we predict indirect interactions from quantitative food webs? ‐ an experimental approach. Journal of Animal Ecology, 80, 108–118. 20796204 10.1111/j.1365-2656.2010.01744.x

[jane13768-bib-0064] Terborgh, J. W. (2015). Toward a trophic theory of species diversity. Proceedings of the National Academy of Sciences of the United States of America, 112, 11415–11422. 26374788 10.1073/pnas.1501070112 PMC4577191

[jane13768-bib-0065] Tilman, D. (2007). Resource competition and plant traits: A response to Craine et al. 2005. Journal of Ecology, 95, 231–234.

[jane13768-bib-0066] Wang, W. C. (1974). Effect of turbidity on algal growth. State of Illinois Circular.

[jane13768-bib-0067] Wittmer, H. U. , Serrouya, R. , Elbroch, L. M. , & Marshall, A. J. (2013). Conservation strategies for species affected by apparent competition. Conservation Biology, 27, 254–260. 23282104 10.1111/cobi.12005

[jane13768-bib-0068] Wootton, J. T. (1994). The nature and consequences of indirect effects in ecological communities. Annual Review of Ecology and Systematics, 25, 443–466.

[jane13768-bib-0069] Wright, A. J. , Wardle, D. A. , Callaway, R. , & Gaxiola, A. (2017). The overlooked role of facilitation in biodiversity experiments. Trends in Ecology & Evolution, 32, 383–390. 28283253 10.1016/j.tree.2017.02.011

