# Supplementary material for: Indirect facilitation between prey promotes asymmetric apparent competition
Source: J Anim Ecol. 2022 Jul 10;91(9):1869–79. doi: 10.1111/1365-2656.13768 (PMC9544837; doi:10.1111/1365-2656.13768)
Supplement: Supplementary file 1 — Figure S1 [file JANE-91-1869-s001.docx]

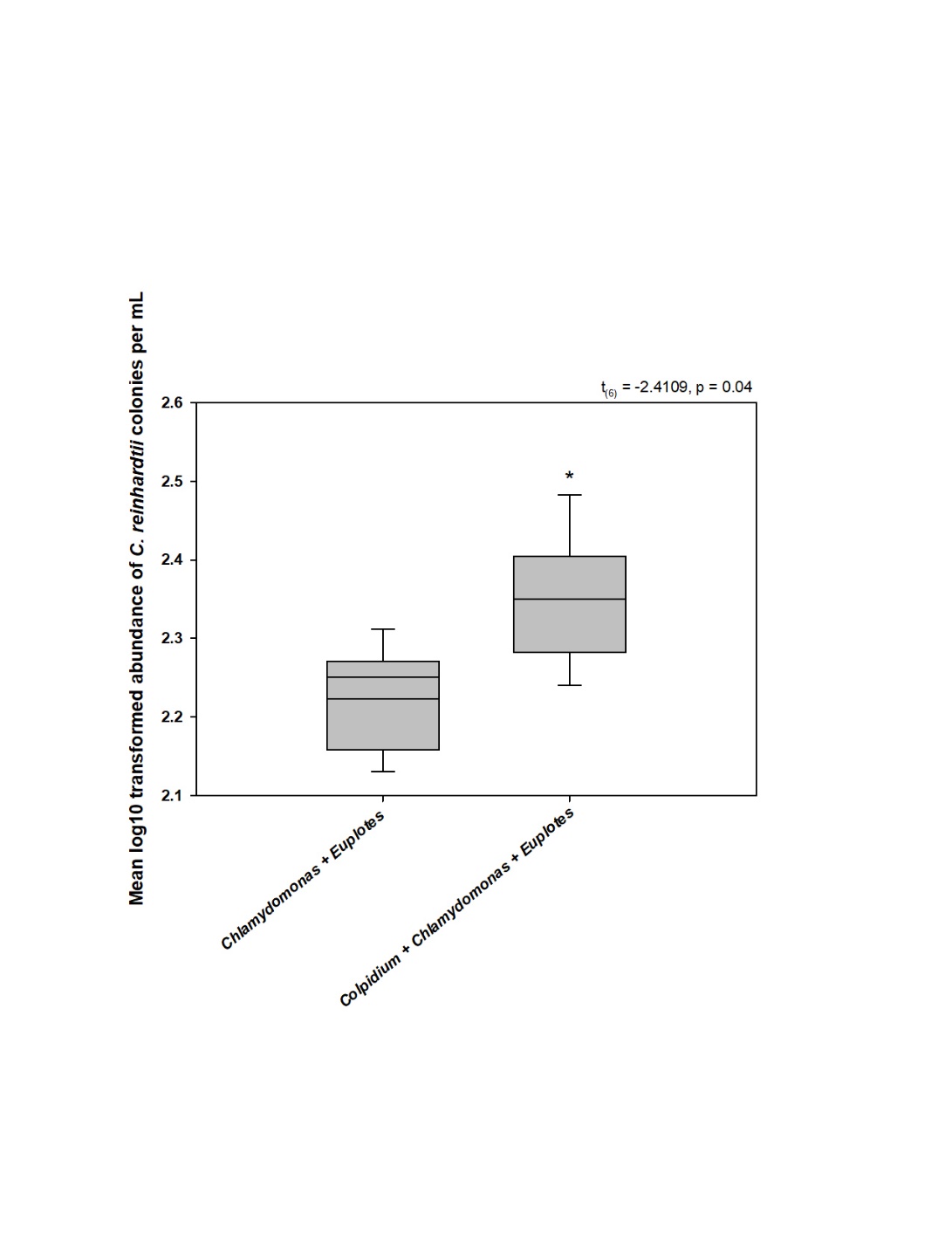


Supplemental Figure 1. Mean log_10_-transformed abundances for *Chlamydomonas* colonies in the two experimental treatments where they were detectable. Asterisk indicates significant difference between treatments (t_(6)_ = -2.4109, p = 0.04). Box plots: middle line, median; box, interquartile range; whiskers, 5th and 95th percentile
